# Worry and Metacognitions as Predictors of Anxiety Symptoms: A Prospective Study

**DOI:** 10.3389/fpsyg.2017.00924

**Published:** 2017-05-31

**Authors:** Truls Ryum, Leif Edward Ottesen Kennair, Odin Hjemdal, Roger Hagen, Joar Øveraas Halvorsen, Stian Solem

**Affiliations:** ^1^Department of Psychology, Norwegian University of Science and TechnologyTrondheim, Norway; ^2^St. Olavs Hospital, Trondheim University HospitalTrondheim, Norway

**Keywords:** metacognition, worry, anxiety, prospective, riskfactor

## Abstract

Both worry and metacognitive beliefs have been found to be related to the development of anxiety, but metacognitive theory (Wells and Matthews, 1994; Wells, 2009) suggest that metacognitive beliefs may play a more prominent role. The aim of the present prospective study was to examine whether worry, metacognitive beliefs or the interaction between worry and metacognitive beliefs, were the best predictor of anxiety over time, utilizing a longitudinal, prospective study design. An undergraduate student sample (*N* = 190) was assessed on measures of worry (PSWQ), metacognitive beliefs (MCQ-30) and anxiety (BAI) at three points in time over a 7-month period. A mixed-model analysis revealed that both worry and metacognitive beliefs predicted development of anxiety, independently of each other, with no indication of an interaction-effect (PSWQ ^*^ MCQ-30). Further, analyses of the MCQ-30 subscales indicated that negative metacognitive beliefs may be particularly important in the development of anxiety. While gender was correlated with worry, gender predicted anxiety beyond the effect of worry. Taken together, the results imply that both worry and metacognitive beliefs play a prominent role for the development of anxiety.

## Introduction

Essentially, metacognition is “thinking about thinking,” and refers to any knowledge or cognitive process involved in the appraisal, monitoring, or control of cognition (Flavell, 1979). Metacognition thus pertains to the stable knowledge about one's own cognitive system and factors that affect the functioning of this system, regulation and awareness of the current state of cognition and appraisal of the significance of thoughts and memories (Wells, 1995, p. 302). Building on this theoretical framework, Wells and Matthews (1994) proposed the Self-Regulatory Executive Function model (S-REF), which provides a conceptualization of metacognitive factors involved in the development and maintenance of anxiety and emotional distress. The basic principle is that emotional distress such as anxiety, comprising emotional, physiological, and cognitive symptoms as operationalized in the Beck Anxiety Inventory (Beck et al., 1988), is maintained by a metacognitive component that regulates cognitive activity and coping behavior. This further promotes extended and perseverative styles of thinking, such as worry, which may give rise to psychological distress, anxiety, depression, and other mental disorders.

The metacognitive model of emotional disorders predicts that the development and maintenance of anxiety, as well as a variety of psychological disorders, are caused and maintained by a cognitive style where one reacts to one's own thoughts with perseverative processing. This may take the form of different types of enduring mental processing, such as worry and rumination, attention on imaginary threats, and the use of maladaptive coping behaviors. This is referred to as the Cognitive Attentional Syndrome (CAS; Wells and Matthews, 1994), which results from positive metacognitive beliefs (e.g., “Worrying helps me cope”) and negative metacognitive beliefs (e.g., “Thoughts are dangerous”) related to mental processing. Fundamentally, this conceptualization suggests that it is the thinking style more than the content of thoughts, which give rise to psychological distress such as anxiety (Wells, 2009).

In this regard, the metacognitive model is at odds with a more traditional cognitive conceptualization, which suggests that anxiety is caused by distortions in cognition (e.g., all or nothing thinking; emotional reasoning) and dysfunctional cognitive beliefs (Beck et al., 1987, 2005). In anxiety, typical beliefs are related to an overestimation of danger as well as an underestimation of coping abilities, such as “The world is an unsafe place” and “I am inadequate and incompetent.” However, whereas both the cognitive- and metacognitive-model agrees that worry is associated with anxiety, as a specific cognitive component involving vigilance and distorted information processing (Craske, 1999; Barlow, 2004), the metacognitive model suggest that it is not necessarily worry *per se* that is problematic, as most people worry (Wells and Morrison, 1994). Rather, it is the metacognitive beliefs that give rise to sustained negative thinking such as worry, which is the driving force behind anxiety (Wells, 2009). Metacognitive theory, then, suggests that increased positive metabeliefs related to potential benefits of worry, combined with negative metabeliefs that worry is uncontrollable and harmful, may be a better predictor of anxiety than worry in itself. It is therefore interesting to explore if metacognitive beliefs predict the development of anxiety over time, even when controlling for the effect of worry.

There have been a number of prospective studies on the relationship between metacognitive beliefs, worry and anxiety, mostly in support of the metacognitive model. For example, Sica et al. (2007) found that negative beliefs about worry predicted worry symptoms after controlling for baseline symptoms of worry and obsessive-compulsive symptoms. Likewise, Thielsch et al. (2015) reported negative metacognitions to predict worry after controlling for trait worry and stress. Moreover, Yilmaz et al. (2011) demonstrated that negative beliefs about worry predicted anxiety after controlling for age, gender, baseline symptoms and life events, and Hjemdal et al. (2013) reported metacognitive beliefs to predict anxiety over a period of 3 months after controlling for age, gender and baseline symptoms. Finally, and at odds with previous research, Ramos-Cejudo and Salguero (2017) found no support for the role of metacognitive beliefs as independent predictors of anxiety over a 3 month period, but negative metacognitive beliefs moderated the relationship between stress and anxiety.

Whereas earlier prospective studies have mostly supported the notion that metacognitive beliefs (especially negative metacognitive beliefs) play a prominent role in the development of anxiety, further prospective studies are needed in order to examine the relationship between worry, metacognitive beliefs, and anxiety in more detail. Of particular note and to the best of our knowledge, no previous prospective study has examined the relative contribution of worry and metacognitive beliefs in the development of anxiety. This is crucial, since the metacognitive model suggests that metacognitive beliefs may play a more prominent role in the development of anxiety, compared to worry (Wells, 2009). Relatedly, metacognitive beliefs may also moderate the effect of worry, which may become problematic only in the context of elevated levels of metacognitive beliefs (see Bailey and Wells, 2015). Lastly, the majority of earlier prospective studies have relied on regression-analysis and a pre-post design, but the use of mixed-model analysis and more frequent assessments is more appropriate when modeling change in anxiety over time. The aim of the present prospective study was therefore to examine whether worry, metacognitive beliefs or the interaction between worry and metacognitive beliefs, were the best predictor of anxiety over time. Based on the S-REF model and the CAS (Wells, 2009), we hypothesized that metacognitive beliefs will predict anxiety better over time, compared to worry, and that the association between worry and anxiety will increase in the context of elevated metacognitive beliefs.

## Methods

### Participants and procedure

A student sample was recruited at a major Norwegian university. Respondents were recruited through lectures, the University's web-site and social media. Inclusion criteria were fluency in Norwegian and minimum 18 years of age. All participation was voluntarily, and participants gave written consent to participate in the study. One hundred and ninety students responded to the questionnaires at all three time points. Mean age was 23.7 years (*SD* = 4.8, range: 19–53). In all, 143 females and 47 males participated. At the end of the study, five gift-vouchers of 1000,- Norwegian krone (≈$118) were drawn from the pool of respondents who contributed data to all three assessment points; (T1) October 2013, (T2) January 2014, and (T3) April 2014. All assessments were completed online, using a unique id-code. Respondents had to respond to all questionnaires at each assessment-point to be able to submit their responses. The study was approved by the Regional committee for medical and health research ethics (ref. nr. 2013/1132).

### Measures

Beck Anxiety Inventory (Beck et al., 1988). This is a 21-item self-report measure assessing anxiety during the last week (e.g., “Unable to relax”). Each item is rated on a 0–3 Likert scale, ranging from “not at all” to “severely,” where higher scores reflect higher levels of anxiety. The BAI has been found to have good psychometric properties, including good reliability and validity (Fydrich et al., 1992). A Norwegian translation was used (Nordhagen et al., 2000), which has been found to have psychometric properties comparable to the original version.

Penn State Worry Questionnaire (Meyer et al., 1990). The PSWQ is a 16-item self-report measure of worry (e.g., “I am always worrying about something”). Each item is rated on a 1–5 Likert scale, ranging from “not at all typical of me” to “very typical of me.” Higher scores indicate higher levels of worry. Psychometric properties of the PSWQ has been reported as good (Stanley et al., 2001; Fresco et al., 2002). The PSWQ has been translated to Norwegian (Pallesen et al., 2006), and found to have adequate psychometric properties in terms of reliability and validity.

Metacognitions Questionnaire 30 (Wells and Cartwright-Hatton, 2004). This is a 30-item self-report questionnaire assessing metacognitive beliefs, which are hypothesized to be risk factors for the development of anxiety and other psychological problems. Each item is rated on a 1–4 Likert scale ranging from “do not agree” to “agree very much.” The questionnaire consists of five subscales [positive beliefs about worry (e.g., “Worrying helps me to solve problems”), negative beliefs about worry (e.g., “When I start worrying I cannot stop”), cognitive confidence (e.g., “I do not trust my memory”), cognitive self-consciousness (“I monitor my thoughts”), and beliefs about the need to control thoughts (e.g., “It is bad to think certain thoughts”)], and higher scores indicate more dysfunctional metacognitive beliefs. The MCQ-30 has shown good psychometric properties including good internal consistency, concurrent- and convergent validity (Spada et al., 2008; Martin et al., 2014; Grotte et al., 2016). The MCQ has been translated to Norwegian and demonstrated good psychometric properties (Grotte et al., 2016).

### Statistics

Bivariate correlation analyses were employed to examine the relationship between the independent variable (BAI) and predictor variables (PSWQ, MCQ-30, MCQ subscales) and demographics (gender, age). Inspection of raw-scores demonstrated that BAI scores were significantly skewed, and these were log-transformed before further analyses were undertaken in a mixed (multilevel) regression analysis (Verbeke and Molenberghs, 2000). A repeated measures design was utilized, with anxiety (BAI) as dependent variable, time of measurement as within-subject factor (0, 1, 2) and assessments of worry (PSWQ) and metacognitions (MCQ-30) as predictors. A random intercept and slope was fitted for each participant. A variance component matrix was used.

## Results

Table 1 presents descriptive statistics for the variables examined.

**Table 1:** Zero-order correlations between worry (PSWQ), metacognitions (MCQ-30), and anxiety (BAI) across three timepoints (*N* = 190).

	**1**	**2**	**3**	**4**	**5**	**6**	**7**	**8**	**9**	**10**	**Gender**	**Age**
1. BAI T1	1	0.78^***^	0.79^***^	0.60^***^	0.62^***^	0.36^***^	0.58^***^	0.28^***^	0.52^***^	0.47^***^	−0.14^*^	−0.07
2. BAI T2		1	0.79^***^	0.49^***^	0.48^***^	0.19^**^	0.44^***^	0.26^**^	0.44^***^	0.38^***^	−0.15^*^	−0.03
3. BAI T3			1	0.51^***^	0.53^***^	0.25^***^	0.49^***^	0.26^***^	0.48^***^	0.40^***^	−0.21^**^	−0.15^*^
4. PSWQ T1				1	0.70^***^	0.44^***^	0.79^***^	0.22^**^	0.56^***^	0.47^***^	−0.25^***^	−0.13
5. MCQ-30 T1					1	0.63^***^	0.84^***^	0.53^***^	0.85^***^	0.74^***^	−0.07	−0.14^*^
6. MCQ-1 T1						1	0.40^***^	0.18^*^	0.47^***^	0.37^***^	−0.16^*^	−0.17^*^
7. MCQ-2 T1							1	0.29^***^	0.68^***^	0.54^***^	−0.10	−0.08
8. MCQ-3 T1								1	0.36^***^	0.12	−0.01	−0.06
9. MCQ-4 T1									1	0.55^***^	0.01	−0.10
10. MCQ-5 T1										1	0.01	−0.12
M (SD)	11.4 (11.9)	10.9 (11.8)	10.4 (11.3)	48.0 (15.3)	55.0 (14.0)	9.8 (3.1)	11.8 (4.7)	10.2 (3.8)	9.8 (3.6)	13.3 (4.3)		23.7 (4.8)
α	0.95	0.94	0.94	0.96	0.91	0.82	0.89	0.83	0.81	0.83		

BAI, Beck Anxiety Inventory; PSWQ, Penn State Worry Questionnaire; MCQ-30, Metacognitions Questionnaire 30; MCQ-1, Positive beliefs about worry; MCQ-2, Negative beliefs about worry concerning uncontrollability and danger; MCQ-3, Lack of cognitive confidence; MCQ-4, Need to control thoughts; MCQ-5, Cognitive self-consciousness; Gender: 1 = male; −1 = female; all correlations are Pearson r, except for Gender which are point-biserial r;

* p ≤ 0.05;

** p < 0.01;

*** *p < 0.001*.

As expected, most study variables were correlated positively at statistically significant levels, except for age. Independent *t*-test demonstrated that females reported significantly higher levels of anxiety at T1, *t*_(188)_ = −1.94, *p* = 0.05, T2, *t*_(188)_ = −2.09, *p* < 0.05, and T3, *t*_(188)_ = −2.93, *p* < 0.01, as well as higher levels of worry (PSWQ) *t*_(188)_ = −3.36, *p* < 0.001. Average-scores were generally below clinical range. Internal consistency (Cronbach's alpha) was excellent (α > 0.9) for all main measures, and good (α > 0.8) for the MCQ-30 subscales.

Table 2 presents a mixed model analysis of BAI across three time points.

**Table 2:** Mixed model analysis using worry (PSWQ) and metacognitions (MCQ-30) as predictors of anxiety (BAI) across three time points.

	**Model 1**		**Model 2**		**Model 3**		**Model 4**	
	**Fixed effects**							
	**Estimate**	***SE***	**Estimate**	***SE***	**Estimate**	***SE***	**Estimate**	***SE***
Intercept	1.08^***^	(0.16)	−0.16	(0.17)	−0.39^*^	(0.16)	−0.82	(0.45)
Time (T1-T3)	−0.02	(0.01)	−0.02	(0.01)	−0.02	(0.01)	−0.02	(0.01)
Gender	−0.20^**^	(0.07)	−0.10	(0.06)	−0.13^**^	(0.05)	−0.13^**^	(0.05)
Age	−0.01	(0.01)	0.00	(0.01)	0.00	(0.00)	0.00	(0.00)
PSWQ T1			0.03^***^	(0.00)	0.01^***^	(0.00)	0.02^*^	(0.01)
MCQ-30 T1					0.01^***^	(0.00)	0.02^**^	(0.01)
PSWQT1 ^*^ MCQ-30 T1							0.00	(0.00)
–2 Log Likelihood	345.20		262.07		224.13		223.11	

Gender: 1 = male, −1 = female; PSWQ, Penn State Worry Questionnaire; MCQ-30, Metacognitions Questionnaire 30; BAI, Beck Anxiety Inventory;

* p < 0.05;

** p ≤ 0.01;

*** *p < 0.001*.

In model 1, the effects of gender and age were first entered. As shown, females reported significantly higher levels of anxiety compared to men. PSWQ was then entered as a predictor in model 2, which significantly predicted anxiety across the three timepoints, *t* = 10.28, *p* < 0.001. Next, in model 3, MCQ-30 was entered as a predictor, which also predicted anxiety, *t* = 6.53, *p* < 0.001. The inclusion of MCQ-30 in model 3 reduced markedly the predictive effect of PSWQ, *t* = 3.64, *p* < 0.001. The *t*-value for PSWQ in model 2 and model 3 were transformed to two correlations *r*^2^ = 0.598, *r*^3^ = 0.256 respectively. These were significance tested yielding a *Z*-score = 4.05, *p* < 0.0001, indicating that the predictive value of PSWQ was significantly reduced when MCQ-30 was included in the analysis. There was no interaction effect of PSWQ and MCQ-30.

Next, a series of mixed-model analyses were computed following a similar procedure as in the forgoing analysis, exchanging the MCQ-30 scores with each of the five MCQ-30 subscales in separate mixed-model analyses. Main effects were found for all MCQ subscales (all *p*'s < 0.001), but model-fit was improved only with subscale MCQ-2 (Negative beliefs), according to the Aikake information criterion (AIC; MCQ-30 = 246.13, MCQ-2 = 239.00, all other MCQ-30 subscales AIC > 246.13). Worry (PSWQ) remained a statistically significant predictor in all analyses, and there was no indication of any interaction-effect between PSWQ and each of the MCQ-30 subscales.

## Discussion

The aim of the present study was to examine whether worry, metacognitive beliefs or the interaction between worry and metacognitive beliefs, were the best predictor of anxiety over time, utilizing mixed-model analysis with repeated assessments. Specifically, we were interested in examining if metacognitive beliefs predicted the development of anxiety over and above that explained by worry. As expected, we found that metacognitive beliefs (MCQ-30) predicted the development of anxiety over time, even when controlling for worry, in accordance with the metacognitive theory (Wells and Matthews, 1994; Wells, 2009). Worry was also a significant predictor of anxiety, and the lack of an interaction-effect (worry ^*^ metacognitive beliefs) indicates that the effects were independent of each other. Whereas overall levels of metacognitive beliefs emerged as a predictor, further analyses revealed that negative beliefs about worry (MCQ-2) was particularly related to the development of anxiety. The results adds to and extends previous research demonstrating metacognitive beliefs to be a prominent temporal antecedent for the development of anxiety (Sica et al., 2007; Yilmaz et al., 2011; Hjemdal et al., 2013), which has important theoretical and clinical implications.

First, worry (PSWQ) was a significant predictor of anxiety across three time points (7 months), which was not surprising given that worry is related to anxiety (e.g., Hong, 2007). That is, higher levels of worry predicted the development of more pronounced anxiety. Worry may be conceptualized as a maladaptive coping strategy (Craske, 1999), typically pertaining to issues of uncertainty and the possibility of negative outcomes (Borkovec et al., 1983). However, worry in itself is not necessarily pathological nor a source of suffering, as most people worry, but only some individuals develop more prominent anxiety (Wells and Morrison, 1994; Wells, 2009). Likewise, individuals higher and lower on worry typically dwell on the same topics, such as relationships, work incompetence, or finances (Tallis et al., 1992). According to the metacognitive model (Wells and Matthews, 1994), metacognitive beliefs may to a larger extent determine whether or not the worry is experienced as problematic and cause anxiety. While positive metacognitive beliefs may increase the amount of worry, it is primarily due to negative metacognitive beliefs that worry and associated anxiety is considered a problem.

Second, as expected, overall levels of metacognitive beliefs (MCQ-30) also predicted development of anxiety over time, after controlling for worry. This is also in accordance with the metacognitive model (Wells, 2009), in which dysfunctional beliefs about cognition is highlighted as prominent risk factors for the development of anxiety. A pattern of thinking dominated by worry, fixation on threat and counterproductive coping strategies such as worry and thought control may unfold, which give rise to a sense of threat, sustained negative thinking, anxiety, and negative emotions. Further analyses demonstrated, in line with most previous studies (Sica et al., 2007; Yilmaz et al., 2011), that negative beliefs about worry as uncontrollable and dangerous, may be particularly important for the development of anxiety. The findings are also in line with previous research, which suggest that metacognitive beliefs (as measured by MCQ-30) are more predictive of symptoms than traditional content measures (e.g., Solem et al., 2009).

Third, the lack of a significant interaction-effect between worry and metacognitive beliefs suggest that the level of metacognitions is not influenced by the level of worry as a predictor of anxiety, and vice versa. This could be due to the fact that the sample reported overall lower levels of anxiety, worry and metacognitive beliefs, and an interaction-effect (worry ^*^ metacognitive beliefs) may at least theoretically be more prominent in populations with more pronounced distress. The use of the PSWQ to assess worry may also have been problematic, as it primarily taps into the unhealthy aspects of worry and not more common worry (Wells and Morrison, 1994). Relatedly, some of the items on the PSWQ bear close resemblance to metacognitive beliefs (e.g., “Once I start worrying, I cannot stop”).

Fourth, the significant effect of gender indicates that males experienced less increase in anxiety over time, compared to females. This is in accordance with the general knowledge on the relationship between gender and anxiety, namely that females are more prone to develop anxiety compared to males (Lewinsohn et al., 1998; McLean et al., 2011). Interestingly, the effect of gender was still significant in the final mixed-model analysis (see Table 2), although gender was positively correlated with worry (see Table 1). This indicates that the gender effect may not be explained by differences in level of worry.

The results demonstrate that higher levels of metacognitive beliefs may be a principal risk factor for the development of anxiety, but future studies are needed in order to examine the relative importance of worry and metacognitive beliefs for the development of anxiety disorders. This also pertains to the temporal relationship between worry and metacognitive beliefs. Moreover, future studies should continue to address the relative importance of various types of metacognitive beliefs for the development of anxiety and disorders, such as positive and negative beliefs. Theoretically (Wells and Matthews, 1994; Wells, 2009), positive metabeliefs about worry may initially be necessary for the individual to engage with this unhelpful strategy, but negative metabeliefs focusing on the uncontrollability and danger of worry may become more prominent at a later developmental stage, as symptoms increase (and worry proves an unsuccessful coping strategy). Lastly, as metacognitive theory suggests that psychological treatment may be facilitated by identifying, challenging and changing metacognitive beliefs (Wells, 2009), rather than dysfunctional cognitions as in traditional cognitive therapy (e.g., Beck et al., 1979; Borkovec, 1994), this assumption should be tested by comparing metacognitive therapy with cognitive therapy in randomized controlled trials for specific disorders.

This study has some limitations that need to be acknowledged. The study utilized a young convenience sample from a student population, who overall reported low levels of worry, metacognitive beliefs, and anxiety. No formal assessment of anxiety diagnosis was undertaken, and to what extent the results may generalize to clinical populations is uncertain and extensions are called for. Future studies need to address these current limitations, especially considering patient populations with higher levels of symptomatology and a true, diagnosed pathology. It might also be relevant to consider effects of age and gender.

In conclusion, the present study indicates that metacognitive beliefs predict the development of anxiety over time, over and above that explained by worry. As this is a prospective, longitudinal study, one may conclude that metacognitive beliefs in general, and negative metabeliefs in particular, are indeed important for the development of anxiety, in accordance with metacognitive theory (Wells, 2009).

## Author contributions

All authors have made significant contributions to design, analyses, and writing of the manuscript.

### Conflict of interest statement

The authors declare that the research was conducted in the absence of any commercial or financial relationships that could be construed as a potential conflict of interest.
